# An Exploratory Biomarker Study of First-Trimester Circulating miRNAs Associated with Later Gestational Diabetes Mellitus

**DOI:** 10.3390/ijms27041920

**Published:** 2026-02-17

**Authors:** Miguel Angel Déctor, Valeria Carmen Macías-González, Adriana Sánchez-García, Armando Hernández-Mendoza, Natalia Martínez-Acuña, Ana María Rivas-Estilla, José Gerardo González-González, María Carmen Barboza-Cerda

**Affiliations:** 1 Facultad de Agronomía, Universidad Autónoma de Nuevo León, Ex Hacienda El Cañada, General Escobedo 66050, Nuevo León, Mexico; miguel.dectorcr@uanl.edu.mx; 2 Independent Researcher, García 66024, Nuevo León, Mexico; 3 Servicio de Endocrinología, Facultad de Medicina y Hospital Universitario “Dr. José Eleuterio González”, Universidad Autónoma de Nuevo León, Mitras Centro, Monterrey 64460, Nuevo León, Mexico; adriana.sanchezga@uanl.edu.mx (A.S.-G.);; 4 Centro de Investigación en Dinámica Celular, Instituto de Investigación en Ciencias Básicas y Aplicadas, Universidad Autónoma del Estado de Morelos, Chamilpa, Cuernavaca 62209, Morelos, Mexico; ahm@uaem.mx; 5 Facultad de Medicina Mexicali, Universidad Autónoma de Baja California, Centro Cívico, Mexicali 21000, Baja California, Mexico; naty.am@gmail.com; 6 Departamento de Bioquímica y Medicina Molecular, Facultad de Medicina y Hospital Universitario “Dr. José Eleuterio González”, Universidad Autónoma de Nuevo León, Av. Francisco I. Madero s/n y Dr. Eduardo Aguirre Pequeño, Mitras Centro, Monterrey 64460, Nuevo León, Mexico

**Keywords:** gestational diabetes mellitus, circulating microRNAs, early pregnancy, insulin secretion, insulin signaling

## Abstract

Gestational diabetes mellitus (GDM) develops silently during early pregnancy, yet its earliest circulating molecular signatures remain poorly defined. In this exploratory biomarker study, we characterized first-trimester circulating microRNA (miRNAs) associated with later GDM using a pool-based small RNA sequencing approach. Using a systematic and unbiased sequencing strategy with locus-level miRNA resolution, we profiled the first-trimester plasma miRNome and prioritized a set of 18 mature miRNAs from among 255 detected species. Set-level functional enrichment analyses based on curated and predicted miRNA–target interactions derived primarily from cellular and tissue-based studies showed annotation-based convergence on pathways related to Ca^2+^ homeostasis, glucagon–insulin regulatory circuits, and PI3K–AKT signaling. Network analysis indicated coordinated associations among these miRNAs and shared target pathways involved in insulin secretion and insulin sensitivity. Key contributors—including miR-29a-3p, miR-29c-3p, miR-146a-5p, let-7a-5p, and miR-182-5p—were linked, through in silico target annotation, to central metabolic regulators such as *PTEN*, *PIK3R1*, *AKT1*, *AKT2*, and components of Ca^2+^ signaling (*ATP2A2*, *CALM1/3*, *ITPR1*, *RYR2*). These circulating miRNAs should be interpreted primarily as biomarkers reflecting coordinated metabolic states rather than as direct causal mediators. Most identified miRNAs have not been previously reported in the context of first-trimester GDM, supporting the exploratory and hypothesis-generating nature of this circulating miRNA signature in early gestational metabolic research.

## 1. Introduction

Gestational diabetes mellitus (GDM) is a frequent pregnancy complication associated with short- and long-term adverse outcomes for both mother and offspring, including hypertensive disorders, macrosomia, neonatal metabolic instability, and increased lifetime risk of type 2 diabetes [1,2,3]. Despite its clinical relevance, GDM is typically diagnosed at 24–28 gestational weeks, leaving limited time for effective interventions. The identification of early, minimally invasive biomarkers associated with subsequent GDM, before overt dysglycemia emerges, remains an important unmet need in clinical research.

Circulating microRNAs (miRNAs) have emerged as promising candidates for early detection because they are stable in plasma, can be measured with high analytical sensitivity, and may reflect underlying physiological states at the tissue level [4,5]. Several studies have reported that women who later develop GDM exhibit differential expression of specific plasma or serum miRNAs already in the first or early second trimester. However, no consensus has emerged regarding which miRNAs represent the most reliable early biomarkers. For example, decreased levels of miR-29a, miR-132, and miR-222 have been reported in early second-trimester serum, whereas five plasma miRNAs—miR-16-5p, miR-17-5p, miR-19a-3p, miR-19b-3p, and miR-20a-5p—were found to be upregulated by next-generation sequencing [6,7]. Other studies have described different sets, including miR-21, miR-155, miR-223, miR-518d-3p, miR-29a, and members of the let-7 family [8,9,10,11,12,13]. Only a few miRNAs, such as miR-29a, have been reported recurrently across two or more independent cohorts. Notably, the reported directionality of miR-29a varies across studies and appears to depend on gestational age and specimen type; moreover, miR-29a has been detected in third-trimester serum, where its combined assessment with miR-29b has been proposed as a diagnostic biomarker for gestational diabetes mellitus, suggesting a gestational stage-dependent behavior of the miR-29 family.

This lack of agreement is likely multifactorial: studies differ markedly in sample type (serum vs. plasma), gestational age at sampling, ethnic and metabolic background of participants, RNA isolation strategies, normalization procedures, and—critically—the technology used for miRNA measurement (TaqMan Low-Density Array [TLDA], microarray, qRT-PCR panels, or limited-depth sequencing). As a result, most published signatures comprise only a small number of detectable miRNAs, and the overlap between studies remains limited despite similar clinical phenotypes.

Given this limited reproducibility and the restricted dynamic range of targeted platforms, it remains unclear whether the full landscape of early-pregnancy circulating miRNAs associated with GDM has been adequately captured. To address this gap, we implemented a high-depth small RNA-sequencing strategy applied to first-trimester plasma, designed to detect both high-abundance and low-frequency circulating miRNAs. Our aim was to identify early circulating miRNA biomarkers associated with later GDM and to explore their potential biological relevance through integrative, set-level in silico analyses of experimentally supported miRNA–target interactions and enriched biological pathways.

## 2. Results

### 2.1. RNA Sequencing

Two independent small RNA sequencing runs were conducted to generate the final dataset. Libraries were pooled equimolarly (4 nM) with an expected yield of approximately 10 million reads per library; however, the first run yielded lower sequencing depth than anticipated. Sequencing depth increased in the second run, in which the n-APO library reached the target coverage (>10 million reads). Across both runs, APO libraries consistently produced higher total read counts, whereas control libraries showed lower yields. These differences reflect technical variability in library performance and sequencing depth and do not represent biological replication.

Quality control of raw FASTQ files using FastQC demonstrated uniformly high per-base quality (QC > 24 for nucleotides 5–75), with no evidence of base-calling bias or sequence deterioration. Adapter trimming retained reads between 15 and 65 nt, consistent with the expected size range of small RNAs. These metrics indicated that reduced yield in the first run was not attributable to read quality or technical artifacts.

Small RNA diversity varied between sequencing runs and did not scale proportionally with total read depth. In the first run, miRNA diversity broadly paralleled sequencing yield, whereas in the second run this relationship was not maintained, indicating that increased depth did not necessarily translate into higher molecular complexity. Mapping to GRCh38 and annotation with miRBase confirmed that the second run improved overall miRNA detection across all groups and was therefore used as the basis for downstream analyses.

### 2.2. First-Trimester Circulating miRNA Profile

Across the three first-trimester plasma libraries—controls with normal OGTT and no adverse outcomes (CON), GDM without adverse pregnancy outcomes (n-APO), and GDM with adverse pregnancy outcomes (APO)—a broad diversity of circulating miRNAs was detected. Using a minimum threshold of ≥1 read in at least one group, a total of 273 distinct miRNAs were identified, representing approximately 14% of the 1918 mature miRNAs annotated in miRBase (July 2021).

The n-APO group exhibited the greatest miRNA diversity (237 miRNAs, 86%), followed by CON (208 miRNAs, 76%) and APO (121 miRNAs, 44%). This group-wise pattern was preserved at higher abundance thresholds. At ≥5 reads, APO retained 94% of its detected miRNAs, compared with 92% in n-APO and 89% in CON. At ≥100 reads, retention was 60% in APO, 49% in n-APO, and 34% in CON, indicating differences in the distribution of highly abundant species across pooled libraries.

Fold-change estimates were obtained for 216 miRNAs in the CON–APO contrast and for 251 miRNAs in the CON–n-APO contrast. After consolidation of both contrasts, fold-change values were available for 255 miRNAs detected across all three pooled groups. Following exclusion of miRNAs with <10 reads in the n-APO group (the library with the highest sequencing depth) and removal of duplicated mature forms, a final high-confidence first-trimester circulating miRNA profile comprising 255 miRNAs was defined for all downstream analyses.

### 2.3. Differential Expression Analysis and Refinement of Candidate miRNAs

Visualization of the relative abundance of all 255 miRNAs across the APO and n-APO subgroups relative to controls provided an overview of the first-trimester circulating miRNA landscape. To focus on expression differences more likely to be informative in plasma, a |mean log_2_FC| ≥ 1.5 threshold was applied, reducing the candidate set to 202 miRNAs distributed as follows:

- 106 miRNAs showing the same direction of change in both APO and n-APO;
- 51 miRNAs displaying differential patterns between the two subgroups;
- 1 miRNA exclusively increased in n-APO;
- 37 miRNAs exclusively decreased in n-APO;
- 7 miRNAs exclusively decreased in APO.

**Figure 1:**
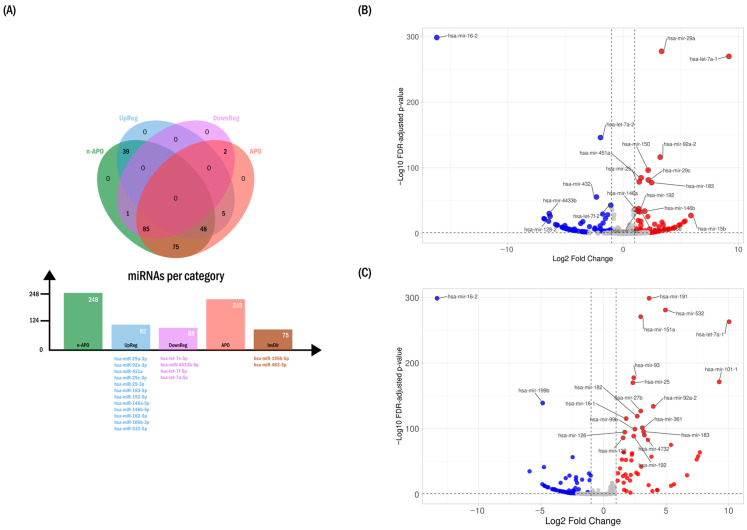
Differential expression of circulating microRNAs (miRNAs) between study subgroups. (**A**) Directional distribution and prioritization of circulating miRNAs. The Venn diagram summarizes the number and direction of circulating miRNAs detected in pooled plasma from the control (CON), GDM without adverse pregnancy outcomes (n-APO), and GDM with adverse pregnancy outcomes (APO) groups (≥10 reads). Representative miRNAs within each expression-direction category are shown below the corresponding bars using matching colors and were prioritized based on Manhattan distance ranking and fold-change patterns (see Table 1). InvDir denotes miRNAs exhibiting inverted directionality between the APO and n-APO groups. (**B**) Volcano plots illustrating fold-change patterns of miRNA abundance in the n-APO pooled group relative to controls. Red dots indicate upregulated miRNAs, whereas blue dots indicate downregulated miRNAs. Dashed lines indicate fold-change thresholds. (**C**) Volcano plots illustrating fold-change patterns of miRNA abundance in the APO pooled group relative to controls. Red dots indicate upregulated miRNAs, whereas blue dots indicate downregulated miRNAs. Dashed lines indicate fold-change thresholds.

These patterns defined seven distinct expression categories, as illustrated in the Venn diagram (Figure 1A).

Applying additional stringency through FDR-adjusted significance (edgeR, FDR < 0.05) together with a log_2_FC threshold of ±1.5 (Figure 1B,C) further reduced the candidate set to 200 miRNAs:

- 107/133 miRNAs showed consistent directionality in both case subgroups;
- 43/47 miRNAs were uniquely altered in one subgroup;
- 50/75 miRNAs displayed opposite directions of change between subgroups.

Using Manhattan distance ranking (VolcaNoseR) together with the filtering criteria described in the Methods, we refined the set of 200 candidate miRNAs, prioritizing those from the n-APO subgroup, which provided the most stable abundance estimates due to higher sequencing depth. This refinement addressed redundancies arising from highly similar mature miRNA sequences derived from paralogous genomic loci. When two precursors could generate the same mature product, raw sequence inspection and relative abundance were used to assign reads to the most likely genomic origin.

During this process, several candidates were excluded, including five paralogous entries among the top 36 Manhattan-distance ranks (MIR16-1, MIR16-2, MIRLET7A1, MIR4433A, and MIRLET7F1), as well as additional miRNAs deprioritized because of low abundance or distant positioning in the APO-based ranking (miR-150, miR-451b, miR-432, miR-148a, miR-15b, miR-128-2, miR-106a, miR-23a, miR-125b-1, miR-99b, miR-181a-1, miR-200c, miR-221, and miR-92a).

This refinement yielded a final set of 18 miRNAs with consistent representation in the APO subgroup, extending to miR-532 (ranked third in APO), which were selected for downstream functional and network analyses. These miRNAs are summarized in Table 1 and displayed beneath the expression bars in Figure 1A.

### 2.4. Targeted qPCR Assessment of Circulating miRNAs

Of the 179 miRNAs assessed by the array platform, 145 (81%) were detected with high precision (Ct ≤ 35) in both case subgroups. Detection categories were distributed as follows: nc/nc: 62% (n-APO) and 75.9% (APO); nc/A or A/nc: 1.1% and 16.2%; A/A: 3.9%; and lower-precision categories B/A (4%), B/B (14%), and C/B (1%).

Among the assayed miRNAs, 88 (49%) reached the ±2.0 fold-regulation cutoff in at least one of the two case subgroups. Of these, 73 met the cutoff in the n-APO subgroup and 25 in the APO subgroup, with partial overlap between subgroups.

Within this set of 88 differentially regulated miRNAs, eight displayed the same direction of change in both n-APO and APO: hsa-miR-200a-3p, hsa-miR-136-3p, and hsa-miR-1-3p were increased, whereas hsa-miR-421, hsa-miR-143-3p, hsa-miR-208a-3p, hsa-let-7a-5p, and hsa-miR-142-3p were decreased. The remaining assayed miRNAs did not exceed the ±2.0 fold-regulation cutoff in either subgroup.

Of the 20 RNA-seq-prioritized miRNAs identified prior to final refinement, 15 (75%) were represented in the array panel and were evaluable (Table 1). Assessment was not possible for hsa-miR-183-5p, hsa-miR-4433b-5p, hsa-miR-182-5p, hsa-miR-196b-5p, or *MIR16-2*. Among evaluable candidates, seven did not surpass the ±2.0 cutoff, four reached the cutoff only in n-APO, and two showed expression patterns opposite to those observed by RNA sequencing.

Partial discordance between RNA-seq and qPCR was expected and likely reflects platform-specific sensitivity, differences in dynamic range, and the use of pooled samples for sequencing. Accordingly, this qPCR assessment should be interpreted as an orthogonal technical confirmation rather than as independent biological validation. hsa-let-7a-5p (derived from *MIRLET7A2*) was the only miRNA meeting all predefined concordance criteria, showing consistent downregulation with high-precision detection.

### 2.5. Disease-Related Similarity Analysis Using TAM

**Figure 2:**
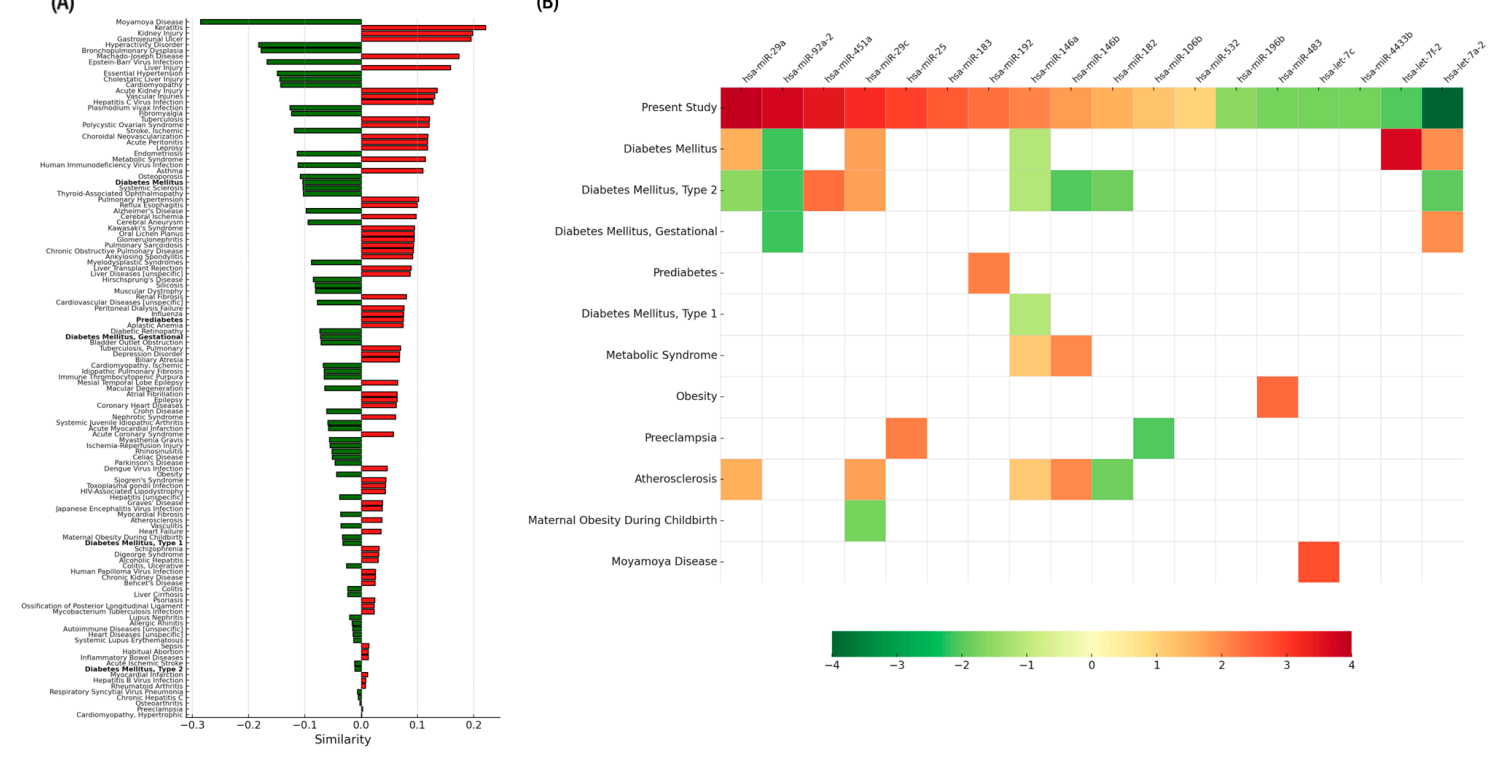
Set-based similarity analysis of the final 18-miRNA signature across disease-associated miRNA profiles. (**A**) Similarity plot generated using TAM 2.0 and edited only to highlight diabetes-related phenotypes in bold, without modifying the original ranking or similarity values. The plot summarizes similarity scores between the collective first-trimester circulating miRNA pattern identified in this study and curated disease-associated miRNA annotations compiled in TAM, which are largely derived from tissue-based expression data. (**B**) Condensed TAM-derived heatmap reordered to display the 18 evaluated miRNAs at the top. This panel represents a composite visualization integrating the 18-miRNA circulating signature identified in this study with disease-associated miRNA profiles curated in TAM. The first row corresponds to the 18-miRNA signature from this study, ordered according to statistical prioritization and expression direction to facilitate comparison with disease-associated profiles. For this first row, the color gradient is a relative and visualization-oriented scale based on statistical significance and direction of expression and does not correspond to the quantitative or relative expression gradients used by TAM for disease-associated profiles. Colors follow a conventional red/green scheme to indicate directionality and are not intended to imply quantitative comparability between datasets. The heatmap illustrates overlap between the circulating miRNA set and disease-associated miRNA annotations, as well as differences in expression direction across profiles.

When first-trimester circulating miRNA profiles were compared with disease-associated signatures largely derived from tissue-based expression data, low or negative similarity scores were observed for diabetes-related categories, including gestational diabetes mellitus, with differences in both the number and the direction of deregulated miRNAs across profiles. In this context, analysis of the 18-miRNA set using TAM (Figure 2A) showed that, among 122 disease profiles, diabetes-related categories ranked at positions 29 (diabetes mellitus), 53 (prediabetes), 56 (gestational diabetes mellitus), 92 (Type 1 diabetes mellitus), and 114 (Type 2 diabetes mellitus). Except for prediabetes, which displayed a positive similarity value, all other diabetes categories exhibited negative similarity values.

The corresponding heatmap (Figure 2B) detailed miRNA overlap with these conditions. Diabetes mellitus shared six miRNAs with the present dataset (miR-29a, miR-92a-2, miR-29c, miR-146a, let-7f-2, let-7a-2), Type 2 diabetes shared eight, gestational diabetes shared two, prediabetes shared one (miR-192), and Type 1 diabetes shared one (miR-146a). For most categories, the direction of expression differed from that observed in our cohort; only a minority of overlapping miRNAs showed concordant directionality.

For comparison, metabolic syndrome (rank 27) shared two miRNAs (miR-146a and miR-146b), both showing the same direction of expression as in our dataset.

### 2.6. Landscape of miRNA–Target Interactions

Integration analyses focused on the final set of 18 mature miRNAs. Experimentally supported miRNA–target interactions were retrieved from miRTarBase (Supplementary Table S4). This curation revealed a non-uniform distribution of interactions, with *PTEN* and *IL6* each annotated as targets of six miRNAs, followed by *CCND2* and *BCL2* with five annotated regulators. Additional multi-targeted genes included *KLF4*, *CDKN1A*, *ITGB1*, *DICER1*, and *MYC* (each targeted by four miRNAs; Supplementary Table S3).

On the miRNA side, the largest numbers of annotated targets corresponded to miR-29a-3p (57 targets), miR-29c-3p (43), miR-146a-5p (28), let-7a-5p (25), and miR-182-5p (22). These highly connected miRNAs accounted for a substantial fraction of the network-level convergence observed in subsequent analyses.

Together, these interactions outline an annotation-based interaction structure in which a subset of highly connected miRNAs maps onto genes involved in metabolic, inflammatory, and insulin-related pathways, forming the basis for the pathway-level patterns described below.

### 2.7. Enriched Functional Pathways Associated with First-Trimester miRNAs

**Figure 3:**
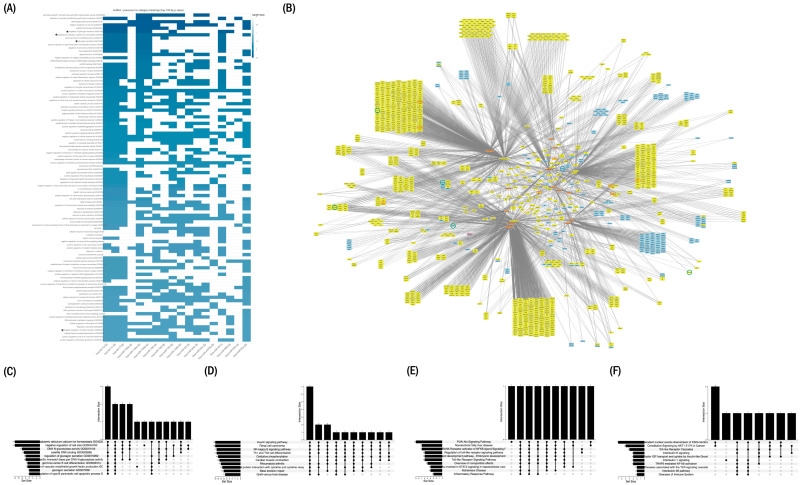
Set-level functional enrichment and annotation-based network analysis of the final 18-miRNA signature. (**A**) miRNA–pathway association heatmap generated with miEAA 2.1 (expert mode; adjusted FDR < 0.05) showing the top 100 enriched categories across KEGG, Reactome, WikiPathways, and Gene Ontology (biological process), based on miRTarBase-derived target annotations. Selected categories of interest within the top 100 are indicated with an asterisk (e.g., regulation of glucagon secretion, endoplasmic reticulum calcium ion homeostasis, glucagon secretion, and negative regulation of insulin secretion). (**B**) Annotation-based miRNA–gene–pathway network generated using MIENTURNET (miRTarBase and TargetScan) and visualized in Cytoscape v3.10.4. Nodes are annotated according to pathway membership: genes associated with PI3K–AKT signaling (red outline), Ca^2+^ homeostasis (green outline), and insulin signaling (blue outline). The five miRNAs with the highest number of annotated targets are highlighted in yellow together with their first neighbors. This representation summarizes shared pathway-level annotations and does not imply direct regulatory interactions in vivo. (**C**) UpSet plot showing the top 10 enriched Gene Ontology categories derived from miRTarBase-filtered target genes under the stringent (FDR-corrected) configuration. (**D**) UpSet plot displaying shared and unique enriched KEGG pathways under the permissive (non–FDR-corrected) configuration. (**E**) UpSet plot displaying shared and unique enriched pathways under the permissive configuration. (**F**) UpSet plots displaying shared and unique enriched Reactome pathways under the permissive configuration. In the UpSet plots, vertical bars represent the number of miRNAs shared among the indicated pathway intersections. Black dots denote observed intersections, whereas light gray dots indicate potential intersections without shared enrichment. Horizontal bars on the left represent the size of each pathway set, proportional to the number of miRNAs associated with each pathway.

Functional enrichment of the 18-miRNA set was evaluated using miEAA over-representation analysis (ORA) under two complementary configurations. The more stringent configuration, incorporating FDR correction and miRTarBase-derived Gene Ontology terms, yielded the most reproducible annotation-based enrichment patterns and showed convergence on categories related to endoplasmic reticulum Ca^2+^ homeostasis (GO:0032469), regulation of glucagon secretion (GO:0070092/GO:0070091), and negative regulation of insulin secretion (GO:0046676). These categories exhibited the strongest overlap across the miRNA set and are summarized in the heatmap (Figure 3A) and primary UpSet plot (Figure 3C).

The more permissive configuration, performed without FDR correction, retrieved broader metabolic and signaling categories across KEGG, WikiPathways, and Reactome, including insulin signaling, PI3K–AKT signaling, and AKT1-related modules (Figure 3D–F). Together, both configurations produced complementary enrichment profiles across endocrine, Ca^2+^-related, and insulin-associated annotations.

### 2.8. Integration of miRNAs with Experimentally Validated Target Genes

To further characterize the annotation-based interaction landscape associated with the 18 miRNAs, each miRNA was integrated with its experimentally supported targets curated in miRTarBase (Supplementary Table S4). The miRNAs showed broad target annotation, with higher-ranking molecules—particularly miR-29a-3p (rank 1), miR-29c-3p (rank 4), miR-182-5p (rank 6), and miR-146a-5p (rank 8)—being linked to multiple genes. Across all annotated targets, *PTEN* was the gene associated with the highest number of miRNAs (6 of the 18 miRNAs; Supplementary Table S3).

Restricting the analysis to genes belonging to ORA-significant pathways (Supplementary Table S2) yielded an annotation-based interaction network (Figure 3B). Two major functional groupings were apparent at the level of pathway annotation. The first comprised genes annotated to Ca^2+^ homeostasis, including *CALM1*, *ITPR1*, and *RYR2* (each linked to at least two miRNAs), *CALM3* (three miRNAs), and *ATP2A2* (up to five miRNAs).

The second grouping corresponded to genes annotated to insulin signaling and the PI3K–AKT pathway, including *AKT1*, *AKT2*, *AKT3*, and *PTEN*, all linked to multiple miRNAs. These genes formed the central cluster of the interaction network in Figure 3B, reflecting the subset of targets most consistently shared across enriched pathway annotations rather than direct regulatory relationships.

## 3. Discussion

Early identification of women at increased risk of developing gestational diabetes mellitus (GDM) remains an unmet clinical priority, particularly during the first trimester, when metabolic disturbances are subtle and preventive interventions may still be most effective. Circulating microRNAs (miRNAs) have therefore attracted interest as early biomarkers, as their abundance in plasma may reflect systemic physiological states and tissue-associated molecular processes. In this study, an unbiased small RNA sequencing approach identified a larger initial set of candidate miRNAs meeting nominal fold-change criteria than typically reported in early pregnancy studies in early pregnancy. Whereas earlier studies [6,7,8,12] typically highlighted only a limited number of candidates, the present dataset initially yielded more than 200 miRNAs meeting nominal fold-change criteria. Through a combination of statistical prioritization, abundance-based ranking, and biological plausibility, this landscape was refined to a focused set of 18 mature miRNAs (Table 1), which together constitute a coherent circulating signature detectable in early pregnancy.

Although the primary aim of this study was biomarker identification, integrative in silico analyses based on curated (miRTarBase) and predicted (TargetScan) miRNA–target interactions, together with pathway-level enrichment (TAM, miEAA, and MIENTURNET), consistently converged on pathways related to Ca^2+^ homeostasis, insulin secretion, and PI3K–AKT signaling (Figure 3). This convergence reflects internal coherence of the identified miRNA set rather than direct evidence of biological regulation. Several of the highest-ranking miRNAs were annotated to genes implicated in glucose metabolism and insulin signaling, including *PTEN*, *PIK3R1*, *AKT1*, *AKT2*, *ITPR1*, *RYR2*, and *MAPK1*. The repeated annotation of these genes across multiple miRNAs suggests that the circulating miRNA signature maps onto established metabolic pathways, providing biological plausibility for the observed associations.

Among the prioritized miRNAs, miR-29a-3p emerged as a prominent contributor. It is one of the few miRNAs recurrently reported in independent GDM cohorts [6,7] and displays an extensive repertoire of experimentally supported targets. Its increased abundance in first-trimester plasma is consistent with prior reports linking miR-29 family members to glucose metabolism and insulin signaling in cellular and animal models. Importantly, previous studies have documented divergent expression patterns for miR-29 family members later in gestation, with reduced levels reported during the second and third trimesters [6]. This apparent discrepancy likely reflects gestational stage-dependent behavior rather than true biological inconsistency.

In this context, the elevated first-trimester levels of miR-29a observed here may represent an early adaptive or compensatory molecular signal that precedes the altered expression patterns reported at later stages of pregnancy. Taken together, these findings support a view of miR-29a as a temporally dynamic circulating biomarker rather than a static indicator of disease status, with its diagnostic relevance depending on the gestational window under investigation.

Other contributors—including miR-29c-3p, miR-146a-5p, miR-182-5p, and members of the let-7 family—further reinforced pathway-level coherence through shared annotation to Ca^2+^ handling and PI3K–AKT signaling pathways. Notably, let-7a-5p ranked highly in the Manhattan distance-based prioritization and showed reduced abundance in first-trimester GDM. Decreased circulating levels of let-7 family members have also been reported in type 2 diabetes mellitus and shown to normalize following glycemic control [15], supporting their broader relevance across metabolic states characterized by impaired glucose homeostasis. Collectively, these miRNAs define a coordinated circulating pattern rather than isolated markers.

Comparisons with other first-trimester studies highlight the context-dependent nature of circulating miRNA signatures. For example, Juchnicka et al. reported increased miR-16-5p, miR-142-3p, and miR-144-3p in first-trimester serum using a NanoString-based platform [16]. The limited overlap with the present dataset likely reflects methodological differences, including analytical platforms, normalization strategies, and selection thresholds, rather than true biological disagreement.

The TAM-based disease similarity analysis showed low or negative similarity scores with diabetes-related categories, including gestational diabetes mellitus. Rather than implying mechanistic opposition, this pattern reflects differences in both the number and the direction of deregulated miRNAs across profiles, as well as the forced comparison between first-trimester circulating miRNA signatures identified in the present study versus disease-associated profiles in TAM that are largely derived from tissue-based expression data. Moreover, this finding is consistent with the reported heterogeneity of circulating miRNA results in gestational diabetes mellitus across primary studies, likely driven by variation in gestational age, biological matrices, and analytical platforms. Altogether, these considerations support a cautious interpretation of TAM similarity rankings and indicate that the first-trimester circulating miRNA patterns observed in this study differ from those reported in overt or later-stage diabetic states.

A notable feature of the present miRNA set was the recurrent annotation of *PTEN* as a target of multiple miRNAs (miR-106b-3p, miR-182-5p, miR-25-3p, miR-29a-3p, miR-29c-3p, and miR-92a-3p). *PTEN* is a central negative regulator of PI3K–AKT signaling, and modest reductions in its expression have been associated with increased insulin sensitivity in experimental systems [17,18]. The convergence of several circulating miRNAs on *PTEN* suggests that early pregnancy may be accompanied by molecular adjustments affecting PI3K–AKT pathway components. However, these annotations should be interpreted cautiously, as they derive from cellular and tissue-based experimental evidence and do not demonstrate functional effects of circulating miRNAs in vivo.

A similar pattern was observed for *FOXO1*-associated pathways. Several miRNAs in the present set were annotated to *FOXO1* or its upstream regulators, consistent with known roles of *FOXO1* in hepatic glucose production and insulin resistance [19]. These associations further support the biological coherence of the circulating miRNA signature but do not imply direct regulatory action by circulating miRNAs.

Pathway enrichment analyses reinforced Ca^2+^-dependent insulin secretion as a recurrent biological theme. Ca^2+^ homeostasis is a sensitive indicator of β-cell function, and dysregulation of Ca^2+^ signaling has been implicated in early β-cell stress and impaired insulin secretion [20]. Broader pathway libraries additionally highlighted PI3K–AKT signaling, a central axis of insulin action. Across multiple annotation databases, these pathways repeatedly emerged among the enriched categories, supporting their plausibility as early metabolic correlates of GDM.

**Figure 4:**
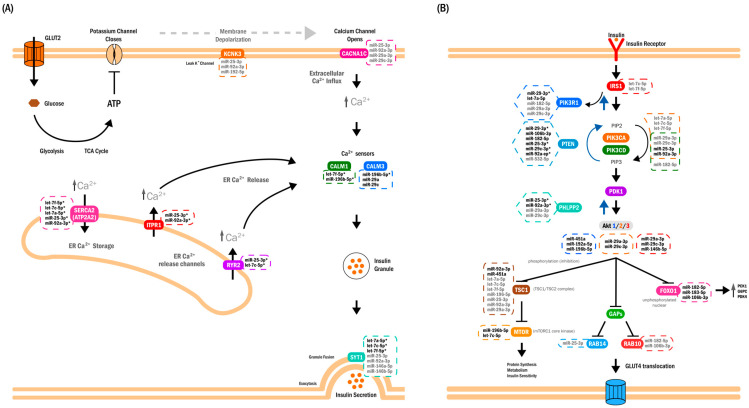
Annotation-based synthesis of pathways associated with the 18-miRNA circulating signature. (**A**) Conceptual schematic of Ca^2+^ handling and excitation–secretion coupling in pancreatic β-cells, included to contextualize enrichment and miRNA–target annotation results. Depolarization activates voltage-dependent Ca^2+^ channels, allowing Ca^2+^ influx, and additional Ca^2+^ release from endoplasmic reticulum stores occurs via ITPR1 and RYR2. The resulting increase in cytosolic Ca^2+^ is sensed by CALM1/CALM3 and associated with SYT1, a Ca^2+^-dependent vesicle-fusion protein located on insulin granules. ATP2A2 (SERCA2) mediates Ca^2+^ re-uptake into the endoplasmic reticulum. Gene symbols indicate miRNA–target annotations identified in this study. CACNA1C is shown as a predicted target because of its established role in voltage-dependent Ca^2+^ entry during glucose-stimulated insulin secretion. Together, these annotations illustrate how the circulating miRNA set maps onto components of Ca^2+^ homeostasis and insulin secretion pathways, without implying direct regulatory effects in vivo. (**B**) Conceptual schematic of the canonical insulin-signaling cascade in skeletal muscle and adipose tissue, included to summarize pathway-level annotations derived from miRNA–target integration. Insulin binding to its receptor (IR) activates IRS1, PI3K, and AKT2, promoting GLUT4 translocation and glucose uptake. Several circulating miRNAs are annotated to components of this pathway, including both positive and inhibitory regulators (e.g., PTEN, PHLPP2, TBC1D1, RAB10). FOXO1 regulation is also depicted: under normal insulin signaling, AKT phosphorylation promotes FOXO1 nuclear exclusion and reduced transcription of gluconeogenic genes (*PCK1*, *G6PC*, *PDK4*). Reduced abundance of selected miRNAs (e.g., miR-182-5p, miR-183-5p, miR-106b-3p) is shown to indicate their annotation to FOXO1-related nodes. This panel summarizes pathway associations suggested by miRNA–target databases and does not represent a mechanistic model. Annotation key (applies to both panels): bold labels denote experimentally supported miRNA–target interactions curated in miRTarBase; gray labels indicate predicted interactions from TargetScan; bold labels with an asterisk indicate interactions supported by both databases. Black arrows indicate canonical pathway signaling direction, whereas blue arrows denote inhibitory regulatory relationships acting in the opposite direction. Panels are ordered according to enrichment strength, with Ca^2+^ homeostasis/insulin secretion (**A**) showing the strongest convergence of miRNA–target annotations, followed by insulin signaling (**B**).

Integration of miRNA–target annotations revealed a structured interaction network characterized by convergence of multiple miRNAs on shared pathway components (Figure 3B and Figure 4). Such convergence is typical of signaling systems in which coordinated modulation of multiple nodes may influence pathway behavior. Importantly, this network representation should be viewed as an annotation-based synthesis rather than a mechanistic model.

Interpretation of circulating miRNAs requires particular caution. At present, these miRNAs should be regarded primarily as biomarkers reflecting coordinated metabolic states rather than as direct causal mediators. Their tissue of origin remains uncertain, and their presence in plasma does not guarantee functional uptake by target cells. Although the placenta contributes substantially to the circulating miRNA pool [21], the patterns observed here do not fully match established placental miRNA profiles [22], suggesting contributions from multiple maternal and fetal tissues. Moreover, the stability, tissue tropism, and biological activity of circulating miRNAs during pregnancy remain incompletely characterized.

Taken together, the present findings support a hypothesis in which early pregnancy may be accompanied by coordinated changes in endocrine and metabolic signaling that are reflected in a structured circulating miRNA signature. By mapping onto pathways related to insulin secretion, insulin action, Ca^2+^ homeostasis, and gluconeogenesis, this signature provides an annotation-based molecular framework for future hypothesis-driven studies of early gestational metabolic adaptation.

This study has several limitations. First, the use of pooled samples increases detection robustness by emphasizing shared molecular features but precludes assessment of inter-individual variability and subject-level inference. Second, although technical replication was performed, biological replication was not feasible within the pool-based design. Third, circulating miRNA concentrations may vary across gestation, and their stability, half-life, and tissue origin remain incompletely defined, which limits the ability to assign observed changes to specific biological sources or temporal dynamics. Finally, functional interpretations and disease similarity inferences rely on curated and predicted miRNA–target annotations and disease signatures derived primarily from cellular and tissue-based studies, making the comparison with circulating miRNA profiles inherently indirect and potentially context-dependent.

## 4. Materials and Methods

### 4.1. Serum Samples and Pool Formation

The serum samples analyzed in this study were obtained from a protocol approved by the Ethics Committee of the School of Medicine at the Universidad Autónoma de Nuevo León (UANL; EN17-00029, 1 August 2017). Women aged 18–35 years with a singleton pregnancy were recruited during the first trimester after providing written informed consent. At this visit, venous blood was collected, serum was separated by standardized centrifugation, and samples were stored at −80 °C. During the second trimester, all participants completed a 75 g oral glucose tolerance test (OGTT). Gestational diabetes mellitus (GDM) was diagnosed when any glucose value exceeded internationally accepted thresholds. Women with pregestational diabetes, prediabetes, polycystic ovary syndrome, a history of GDM or preeclampsia, multiple pregnancy, or use of medications affecting glucose homeostasis were excluded. Serum samples showing hemolysis or insufficient volume were removed prior to analysis.

A total of 205 serum samples were available (171 controls and 34 GDM cases). To optimize sequencing resources, three independent pools of 10 samples each were prepared. The control pool (CON) included women with normal OGTT results and without maternal–fetal adverse outcomes.

Pooling of serum samples was performed within each clinical subgroup prior to RNA extraction. Equal volumes of individual serum samples were combined to generate subgroup-level pools for downstream small RNA sequencing. While this approach precludes estimation of inter-individual variance, pooling was implemented to enhance detection robustness and reduce within-group biological variability, thereby improving the stability of expression estimates for low-abundance circulating miRNAs, as previously described for RNA sequencing-based studies [23].

GDM cases were divided into two subgroups according to clinical outcomes. The n-APO pool comprised women with GDM who did not present adverse maternal–fetal outcomes. These participants typically exhibited overweight or obesity (by BMI), had first-degree family history of type 2 diabetes in several cases, and showed one or two elevated OGTT values—most frequently from the fasting measurement—while remaining free of obstetric or neonatal complications.

The APO pool consisted of women with GDM who experienced adverse maternal–fetal outcomes, including preeclampsia, macrosomia, or preterm birth. These participants generally had obesity, a positive first-degree family history in multiple cases, and exhibited two or three elevated OGTT values. The presence of adverse outcomes defined this subgroup.

The three pools were matched for general clinical characteristics and were used as the experimental units for RNA extraction and small RNA sequencing. Individual-level clinical information for the 30 women included in the pools is provided in Supplementary Table S1.

### 4.2. RNA Extraction and Small RNA Sequencing

Circulating miRNAs were extracted from serum using the miRNeasy Serum/Plasma Kit (QIAGEN, Hilden, Germany) according to the manufacturer’s instructions. Briefly, 200 μL of serum were lysed with QIAzol Lysis Reagent (5:1 ratio), followed by chloroform addition and phase separation by centrifugation. The aqueous phase containing total RNA was recovered, mixed with 100% ethanol, and loaded onto RNeasy MinElute columns. After sequential washes, total RNA obtained from a combined serum volume of 400 μL was eluted in 14 μL of RNase-free water.

RNA concentration and purity were assessed using 1 μL of each sample on a NanoDrop One/OneC Microvolume UV–Vis Spectrophotometer (Thermo Fisher Scientific, Waltham, MA, USA). RNA purity was evaluated based on A260/A280 ratios (1.8–2.0) to detect protein or phenol contamination and A260/A230 ratios (>2.0) to assess potential contamination with salts or organic compounds.

### 4.3. miRNA Library Preparation

miRNA libraries were prepared using the QIAseq miRNA NGS 12 Index Kit (QIAGEN, Hilden, Germany) following the manufacturer’s protocol. Three libraries corresponding to the experimental groups were generated. In brief, adapters were sequentially ligated to the 3′ and 5′ ends of mature miRNAs, followed by reverse transcription incorporating unique molecular identifiers (UMIs). After purification, libraries were PCR-amplified using a universal 5′ primer and an indexed 3′ primer, and purified using magnetic beads prior to sequencing.

Libraries were quantified fluorometrically, normalized, and pooled in equimolar proportions. The pooled libraries were purified by native 6% polyacrylamide gel electrophoresis for size selection. After DNA visualization, the fraction corresponding to the expected size range for miRNA libraries (approximately 150–180 bp) was excised.

DNA was eluted from the gel, filtered, and concentrated using ultrafiltration devices, and subsequently quantified using the Qubit^®^ dsDNA HS Assay Kit (Thermo Fisher Scientific, Waltham, MA, USA). The purified library pool was adjusted to the required concentration and submitted for Illumina sequencing at the Laboratorio Nacional de Apoyo Tecnológico a las Ciencias Genómicas (LNATCG), Institute of Biotechnology, UNAM, Cuernavaca, Morelos, Mexico.

### 4.4. Sequencing Data Analysis

Small RNA–seq reads (single-end, 75 bp) generated on the Illumina NextSeq 550 platform (Illumina, San Diego, CA, USA) were assessed for base quality, adapter content, and sequence composition using FastQC v0.12.0 (Babraham Bioinformatics, Cambridge, UK). Adapter trimming and size filtering were performed with Cutadapt v3.4 (Marcel Martin, Heidelberg, Germany), retaining fragments between 18 and 55 nucleotides, a range compatible with mature miRNAs, isomiRs, and additional short RNA species expected from the QIAseq miRNA library workflow.

Quality-filtered reads were aligned to the human reference genome GRCh38 using ShortStack v3.8.5 (Pennsylvania State University, University Park, PA, USA), which employs Bowtie for small-RNA-optimized placement. To ensure annotation precision and prevent ambiguity arising from overlapping small RNA clusters, no de novo locus discovery was enabled. Instead, read assignment was constrained to a curated miRNA locifile derived from miRBase v22 [24], containing predefined genomic intervals for each mature miRNA (e.g., chr1:17369–17436 → hsa-mir-6859-1; chr1:9151668–9151777 → hsa-mir-34a). Resulting alignment (BAM) files were indexed using SAMtools (Wellcome Trust Sanger Institute, Hinxton, UK) and subjected to standard quality inspection.

Raw miRNA count data generated by ShortStack [25] were analyzed using edgeR (version 3.24.3) [26]. Library sizes were normalized using the trimmed mean of M values (TMM) method [27], and differential expression was assessed by fitting a negative binomial generalized linear model to contrast experimental groups. Statistical significance was evaluated using the Benjamini–Hochberg false discovery rate (FDR) correction [28]. miRNAs with |log_2_ fold-change| ≥ 1.5 and FDR < 0.05 were considered differentially expressed and carried forward for functional prioritization. Volcano plots were generated using VolcaNoseR [29], and normalized read counts were used for integrative analyses including similarity profiling, target-gene mapping, and pathway enrichment.

### 4.5. Rationale for Selecting edgeR as the Primary Differential Expression Tool

Although DESeq2 and edgeR were initially evaluated in parallel, edgeR was selected as the primary tool for downstream analyses based on its statistical behavior and its biological relevance to circulating miRNAs. For extracellular miRNAs, abundance is directly linked to putative regulatory capacity: highly expressed miRNAs are more likely to exert measurable post-transcriptional repression, whereas low-copy miRNAs—even when statistically significant—may have limited functional impact.

During preliminary comparisons, DESeq2 tended to assign higher statistical weight to low-abundance miRNAs, prioritizing features that were less aligned with our criteria for functional relevance. In contrast, edgeR emphasized miRNAs with high counts, consistent detection across case groups, or marked depletion in specific clinical subgroups (n-APO or APO), yielding results that better reflected our biological rationale. Although both tools produced comparable fold-change estimates, their significance distributions differed, with edgeR providing a ranking more consistent with abundance-driven relevance and presence–absence patterns.

Accordingly, edgeR enabled a biologically coherent prioritization of differentially expressed miRNAs, focusing on candidates most likely to contribute to GDM-related regulatory networks.

### 4.6. Targeted qPCR Assessment of Circulating miRNAs

To provide a targeted, orthogonal assessment of circulating miRNA abundance, we employed a real-time quantitative PCR (RT–qPCR) approach with the miRCURY LNA™ miRNA Focus PCR Panels (QIAGEN; YAHS-106Y) in combination with the miRCURY LNA™ SYBR^®^ Green Master Mix. Instead of individual serum samples, we used the three previously generated RNA pools (CON, n-APO, APO), each obtained from 10 patients per clinical group as described above.

Purified RNA from each pool (eluted in 14 µL of RNase-free water) was reverse-transcribed using the miRCURY LNA^®^ RT Kit (QIAGEN, HB-2439; optimized for serum/plasma RNA), following the manufacturer’s protocol. The resulting cDNA was mixed with the SYBR Green Master Mix and dispensed into the wells of the PCR array.

qPCR reactions were run on a StepOnePlus™ Real-Time PCR System (Applied Biosystems, Thermo Fisher Scientific, Waltham, MA, USA) using the recommended two-step cycling protocol for 40 cycles. Raw CT values were exported for downstream analysis.

CT values for all miRNAs detected in the three pools were uploaded to the QIAGEN GeneGlobe data analysis portal “https://www.qiagen.com/geneglobe (accessed on 21 July 2022)”. Samples were labeled as “Control Group” (CON) and “Test Groups” (n-APO and APO).

Normalization was performed using the geNorm “Pre-Defined Reference miRNAs Only” strategy, which selected a stable set of reference miRNAs: hsa-miR-93-5p, hsa-miR-191-5p, hsa-miR-423-5p, and hsa-miR-425-5p. A CT cutoff of 35 was applied as the lower limit of detection.

Relative expression was calculated using the ΔΔCT method (ddCT). First, ΔCT was calculated by subtracting the average CT of reference miRNAs from the CT of each target miRNA. Then, ddCT values were computed as:

ΔΔCT = ΔCT(Test) − ΔCT(Control)

Fold-change (FC) values were calculated using the 2^−ΔΔCt^ method, and miRNAs with FC ≥ 2 in either test group were classified as differentially expressed for downstream analyses.

qRT-PCR detection comments (A, B, C, and nc) were assigned automatically by the miRCURY LNA miRNA PCR Data Analysis software (QIAGEN, Hilden, Germany) according to manufacturer-defined Ct-based criteria. Briefly, category A indicates low expression in one group (mean Ct > 30), category B indicates low expression in both groups, category C indicates no detectable expression, and nc indicates reliable detection in both groups.

Principal component analysis (PCA) was evaluated for exploratory purposes but was not retained for the final analysis.

### 4.7. Functional Analyses and Integrative Visualization

#### 4.7.1. Volcano Plots

Volcano plots were generated using VolcaNoseR2 (University Medical Center Utrecht, Utrecht, The Netherlands), based on normalized log_2_ fold-change values and FDR-adjusted *p*-values obtained from edgeR for the comparisons n-APO vs. CON and APO vs. CON. The x-axis represented the log_2_ fold change, and the y-axis represented −log_10_(FDR).

A fold-change threshold of |log_2_FC| ≥ 1 and an FDR threshold of 0.05 (−log_10_FDR ≥ 1.3) were applied. Data points were color-coded according to regulation category, and prominent miRNAs were ranked using the Manhattan distance criterion. Gene names were used as labels for miRNAs meeting the applied thresholds. All plots were generated using standardized axis labels and layout parameters.

#### 4.7.2. Interaction Network Construction

MiRNA–target interaction data were retrieved using MIENTURNET [30], integrating experimentally validated interactions from miRTarBase and predicted interactions from TargetScan. The list of mature miRNAs selected for downstream analysis was entered manually, and the complete set of miRNA–gene interaction pairs was retrieved. The resulting interaction table was exported in .csv format and used to construct integrated miRNA–target matrices and for subsequent visualization steps.

Network construction and rendering were performed in Cytoscape v3.10.4 (macOS) [31]. Attribute-based visual styles were applied uniformly to standardize node appearance and layout across the network.

#### 4.7.3. Functional Set Enrichment and Similarity Analysis (TAM 2.0)

Functional similarity analyses were performed using TAM 2.0 [32] in the Comparison mode. Differentially expressed miRNAs were grouped by direction of expression and entered as separate input lists using the human pre-miRNA (HGNC) format. In this mode, TAM compared each input list against curated disease-associated miRNA sets and generated similarity matrices.

The “Mask cancer-related terms” and “Mask non-standard terms” filters were applied. Output similarity matrices and heatmaps were downloaded for downstream processing and visualization.

#### 4.7.4. Functional Enrichment Analysis (miEAA)

Functional enrichment analyses were conducted using miEAA 2.1 [33] under an Over-Representation Analysis (ORA) framework. Analyses were performed using the annotation filter “Annotations derived over miRTarBase (Gene Ontology)” and Expert Mode to query KEGG, Reactome, and WikiPathways. Statistical significance was assessed using Benjamini–Hochberg FDR correction.

Analyses were performed using collective mode, treating all differentially expressed miRNAs as a single input set. Alternative parameter configurations were also evaluated to generate complementary enrichment outputs used in downstream visualizations.

## 5. Conclusions

In conclusion, this study identified a set of first-trimester circulating miRNAs associated with later gestational diabetes mellitus using an exploratory, pool-based design. This miRNA set shows coherent annotation-based convergence on pathways related to Ca^2+^-dependent insulin secretion, PI3K–AKT signaling, and *FOXO1*-associated metabolic regulation. These findings are consistent with the presence of coordinated molecular patterns detectable in plasma early in pregnancy but should be interpreted as hypothesis-generating rather than predictive or mechanistic. Further studies in individual samples and experimental systems will be required to validate these associations and to determine whether these circulating miRNAs act solely as biomarkers of early metabolic adaptation or also participate in disease-relevant regulatory processes.

## Figures and Tables

**Table 1:** Top 18 significant circulating miRNAs detected in first-trimester plasma from pregnancies that later developed gestational diabetes mellitus.

miRBase Database Entries				Non-Adverse Pregnancy Outcomes (n-APO)			Adverse Pregnancy Outcomes (APO)			**Control Group**	**Fold Regulation** **qRT-PCR Array**			
Stem-Loop ID	HGNC	Mature Sequence ID	Accession	miRNAs Ranking	FC	*p*-Value	miRNAs Ranking	FC	*p*-Value	**Reads ^§^**	**n-APO**		**APO**	
**hsa-mir-29a**	*MIR29A*	hsa-miR-29a-3p	MIMAT0000086	1	3.33	277.49	*	0.88	9.50	269	1.00	nc	−3.22	nc
**hsa-let-7a-2**	*MIRLET7A2*	hsa-let-7a-5p	MIMAT0000062	2	−1.96	146.16	40	−1.02	28.63	3252	**−2.01**	nc	**−3.23**	nc
**hsa-mir-92a-2**	*MIR92A2*	hsa-miR-92a-3p	MIMAT0000092	3	3.21	116.29	10	3.97	133.88	74	**4.07**	**nc**	**1.27**	**nc**
**hsa-mir-451a**	*MIR451A*	hsa-miR-451a	MIMAT0001631	4	1.55	84.76	21	1.61	64.01	859	1.00	nc	**1.26**	nc
**hsa-mir-29c**	*MIR29C*	hsa-miR-29c-3p	MIMAT0000681	5	2.19	81.58	29	2.22	51.72	154	1.00	B	−1.59	B
**hsa-mir-25**	*MIR25*	hsa-miR-25-3p	MIMAT0000081	6	1.40	78.57	8	2.34	170.00	2022	**2.01**	nc	**1.26**	nc
**hsa-mir-183**	*MIR183*	hsa-miR-183-5p	MIMAT0000261	7	2.47	77.46	13	3.20	95.33	88	NI		NI	
**hsa-let-7f-2**	*MIRLET7F2*	hsa-let-7f-5p	MIMAT0000067	8	−1.07	42.90	43	−1.16	31.80	2133	**−2.00**	A	**−1.59**	nc
**hsa-mir-192**	*MIR192*	hsa-miR-192-5p	MIMAT0000222	9	1.36	37.61	16	2.41	88.52	237	−1.01	nc	−1.60	nc
**hsa-mir-146a**	*MIR146A*	hsa-miR-146a-5p	MIMAT0000449	10	1.05	36.88	31	1.31	39.48	750	**1.01**	nc	**1.27**	nc
**hsa-mir-4433b**	*MIR-4433B*	hsa-miR-4433b-5p	MMAT00030413	11	−6.41	30.31	56	−4.71	12.94	84	NI		NI	
**hsa-mir-146b**	*MIR146B*	hsa-miR-146b-5p	MIMAT0002809	12	1.86	33.94	38	2.17	29.46	71	1.01	B	−1.58	B
**hsa-mir-182**	*MIR182*	hsa-miR-182-5p	MIMAT0000259	13	1.28	33.86	10	2.69	119.01	247	NI		NI	
**hsa-mir-106b**	*MIR106B*	hsa-miR-106b-3p	MIMAT0004672	14	1.43	32.98	44	1.74	30.31	154	**2.03**	B	−1.57	B
**hsa-let-7c**	*MIRLET7C*	hsa-let-7c-5p	MIMAT0000064	15	−1.77	29.64	35	−4.76	41.45	263	1.01	nc	−1.59	nc
**hsa-mir-483**	*MIR483*	hsa-miR-483-5p	MIMAT0004761	16	−1.33	28.51	*	0.72	7.71	366	1.04	B	1.27	A
**hsa-mir-196b**	*MIR196B*	hsa-miR-196b-5p	MIMAT0001080	17	−3.49	18.06	*	0.69	2.06	79	NI		NI	
**hsa-mir-532**	*MIR532*	hsa-miR-532-5p	MIMAT0002888	18	1.53	18.73	3	4.94	281.04	73	**2.11**	B	−1.55	B

Ranking based on Manhattan distance metrics for microRNAs (miRNAs), primarily derived from the non-adverse pregnancy outcomes (n-APO) subgroup, after exclusion of five paralogous entries and 13 additional low-priority candidates, resulting in a final set of 18 miRNAs. Bolded entries indicate concordant directionality between RNA-seq log_2_ fold change and fold regulation. *p*-value: FDR-adjusted *p*-values (−log_10_); FC: log_2_ fold change; * miRNAs that did not meet the inclusion criteria for the top 100 Manhattan-distance ranking; ^§^ raw reads from the second RNA-seq of the control group; A, B and nc: qRT-PCR detection comments defined in the Materials and Methods; NI: not included.

## Data Availability

The datasets generated and/or analyzed during the current study are not publicly available due to ethical and privacy considerations but are available from the corresponding author upon reasonable request.

## Supplementary Materials

The following supporting information can be downloaded at https://www.mdpi.com/article/10.3390/ijms27041920/s1.

## Abbreviations

The following abbreviations are used in this manuscript:

GDM	Gestational diabetes mellitus
DM	Diabetes mellitus
T1D	Type 1 diabetes
T2D	Type 2 diabetes
miRNA	MicroRNA
RNA-seq	RNA sequencing
CON	Control
APO	Adverse pregnancy outcomes
n-APO	non-Adverse pregnancy outcomes
Ca^2+^	Calcium ion
PI3K	Phosphatidylinositol 3-kinase
AKT	Protein kinase B
FOXO1	Forkhead box protein O1
PTEN	Phosphatase and tensin homolog
IRS	Insulin receptor substrate
ORA	Over-representation analysis
FDR	False discovery rate
RISC	RNA-induced silencing complex
qPCR	Quantitative polymerase chain reaction
SERCA	Sarco/endoplasmic reticulum Ca^2+^-ATPase (ATP2A2)
KEGG	Kyoto Encyclopedia of Genes and Genomes
GO	Gene Ontology
TAM	Tool for Annotations of human MicroRNAs
miEAA	MicroRNA Enrichment Analysis and Annotation
PCA	Principal component analysis

## References

[1] Martínez-Ibarra A., Martínez-Razo L.D., Vázquez-Martínez E.R., Martínez-Cruz N., Flores-Ramírez R., García-Gómez E., López-López M., Ortega-González C., Camacho-Arroyo I., Cerbón M. (2019). Unhealthy Levels of Phthalates and Bisphenol A in Mexican Pregnant Women with Gestational Diabetes and Its Association to Altered Expression of miRNAs Involved with Metabolic Disease. Int. J. Mol. Sci..

[2] Nevalainen J., Sairanen M., Appelblom H., Gissler M., Timonen S., Ryynänen M. (2016). First-Trimester Maternal Serum Amino Acids and Acylcarnitines Are Significant Predictors of Gestational Diabetes. Rev. Diabet. Stud..

[3] Rasanen J.P., Snyder C.K., Rao P.V., Mihalache R., Heinonen S., Gravett M.G., Roberts C.T., Nagalla S.R. (2013). Glycosylated Fibronectin as a First-Trimester Biomarker for Prediction of Gestational Diabetes. Obstet. Gynecol..

[4] Arroyo J.D., Chevillet J.R., Kroh E.M., Ruf I.K., Pritchard C.C., Gibson D.F., Mitchell P.S., Bennett C.F., Pogosova-Agadjanyan E.L., Stirewalt D.L. (2011). Argonaute2 Complexes Carry a Population of Circulating microRNAs Independent of Vesicles in Human Plasma. Proc. Natl. Acad. Sci. USA.

[5] Chen X., Ba Y., Ma L., Cai X., Yin Y., Wang K., Guo J., Zhang Y., Chen J., Guo X. (2008). Characterization of microRNAs in Serum: A Novel Class of Biomarkers for Diagnosis of Cancer and Other Diseases. Cell Res..

[6] Zhao C., Dong J., Jiang T., Shi Z., Yu B., Zhu Y., Chen D., Xu J., Huo R., Dai J. (2011). Early Second-Trimester Serum MiRNA Profiling Predicts Gestational Diabetes Mellitus. PLoS ONE.

[7] Zhu Y., Tian F., Li H., Zhou Y., Lu J., Ge Q. (2015). Profiling Maternal Plasma microRNA Expression in Early Pregnancy to Predict Gestational Diabetes Mellitus. Int. J. Gynecol. Obstet..

[8] Gillet V., Ouellet A., Stepanov Y., Rodosthenous R.S., Croft E.K., Brennan K., Abdelouahab N., Baccarelli A., Takser L. (2019). miRNA Profiles in Extracellular Vesicles From Serum Early in Pregnancies Complicated by Gestational Diabetes Mellitus. J. Clin. Endocrinol. Metab..

[9] Hocaoglu M., Demirer S., Senturk H., Turgut A., Komurcu-Bayrak E. (2019). Differential Expression of Candidate Circulating microRNAs in Maternal Blood Leukocytes of the Patients with Preeclampsia and Gestational Diabetes Mellitus. Pregnancy Hypertens..

[10] Lamadrid-Romero M., Solís K.H., Cruz-Reséndiz M.S., Pérez J.E., Díaz N.F., Flores-Herrera H., García-López G., Perichart O., Reyes-Muñoz E., Arenas-Huertero F. (2018). Central Nervous System Development-Related microRNAs Levels Increase in the Serum of Gestational Diabetic Women during the First Trimester of Pregnancy. Neurosci. Res..

[11] Pheiffer C., Dias S., Rheeder P., Adam S. (2018). Decreased Expression of Circulating miR-20a-5p in South African Women with Gestational Diabetes Mellitus. Mol. Diagn. Ther..

[12] Tagoma A., Alnek K., Kirss A., Uibo R., Haller-Kikkatalo K. (2018). MicroRNA Profiling of Second Trimester Maternal Plasma Shows Upregulation of miR-195-5p in Patients with Gestational Diabetes. Gene.

[13] Vasu S., Kumano K., Darden C.M., Rahman I., Lawrence M.C., Naziruddin B. (2019). MicroRNA Signatures as Future Biomarkers for Diagnosis of Diabetes States. Cells.

[14] Deng L., Huang Y., Li L., Chen H., Su J. (2020). Serum miR-29a/b Expression in Gestational Diabetes Mellitus and Its Influence on Prognosis Evaluation. J. Int. Med. Res..

[15] Santovito D., De Nardis V., Marcantonio P., Mandolini C., Paganelli C., Vitale E., Buttitta F., Bucci M., Mezzetti A., Consoli A. (2014). Plasma Exosome MicroRNA Profiling Unravels a New Potential Modulator of Adiponectin Pathway in Diabetes: Effect of Glycemic Control. J. Clin. Endocrinol. Metab..

[16] Juchnicka I., Kuźmicki M., Niemira M., Bielska A., Sidorkiewicz I., Zbucka-Krętowska M., Krętowski A.J., Szamatowicz J. (2022). miRNAs as Predictive Factors in Early Diagnosis of Gestational Diabetes Mellitus. Front. Endocrinol..

[17] Morley T.S., Xia J.Y., Scherer P.E. (2015). Selective Enhancement of Insulin Sensitivity in the Mature Adipocyte Is Sufficient for Systemic Metabolic Improvements. Nat. Commun..

[18] Stiles B., Wang Y., Stahl A., Bassilian S., Lee W.P., Kim Y.-J., Sherwin R., Devaskar S., Lesche R., Magnuson M.A. (2004). Liver-Specific Deletion of Negative Regulator Pten Results in Fatty Liver and Insulin Hypersensitivity. Proc. Natl. Acad. Sci. USA.

[19] Kim J.J., Li P., Huntley J., Chang J.P., Arden K.C., Olefsky J.M. (2009). FoxO1 Haploinsufficiency Protects against High-Fat Diet-Induced Insulin Resistance with Enhanced Peroxisome Proliferator-Activated Receptor Gamma Activation in Adipose Tissue. Diabetes.

[20] Sabatini P.V., Speckmann T., Lynn F.C. (2019). Friend and Foe: β-Cell Ca^2+^ Signaling and the Development of Diabetes. Mol. Metab..

[21] Miura K., Miura S., Yamasaki K., Higashijima A., Kinoshita A., Yoshiura K., Masuzaki H. (2010). Identification of Pregnancy-Associated MicroRNAs in Maternal Plasma. Clin. Chem..

[22] Williams Z., Ben-Dov I.Z., Elias R., Mihailovic A., Brown M., Rosenwaks Z., Tuschl T. (2013). Comprehensive Profiling of Circulating microRNA via Small RNA Sequencing of cDNA Libraries Reveals Biomarker Potential and Limitations. Proc. Natl. Acad. Sci. USA.

[23] Assefa A.T., De Paepe K., Everaert C., Mestdagh P., Thas O., Vandesompele J. (2018). Differential Gene Expression Analysis Tools Exhibit Substandard Performance for Long Non-Coding RNA-Sequencing Data. Genome Biol..

[24] Kozomara A., Birgaoanu M., Griffiths-Jones S. (2019). miRBase: From microRNA Sequences to Function. Nucleic Acids Res..

[25] Axtell M.J. (2013). ShortStack: Comprehensive Annotation and Quantification of Small RNA Genes. RNA.

[26] Robinson M.D., McCarthy D.J., Smyth G.K. (2010). edgeR: A Bioconductor Package for Differential Expression Analysis of Digital Gene Expression Data. Bioinformatics.

[27] Robinson M.D., Oshlack A. (2010). A Scaling Normalization Method for Differential Expression Analysis of RNA-Seq Data. Genome Biol..

[28] Benjamini Y., Hochberg Y. (1995). Controlling the False Discovery Rate: A Practical and Powerful Approach to Multiple Testing. J. R. Stat. Soc. Ser. B Stat. Methodol..

[29] Goedhart J., Luijsterburg M.S. (2020). VolcaNoseR Is a Web App for Creating, Exploring, Labeling and Sharing Volcano Plots. Sci. Rep..

[30] Licursi V., Conte F., Fiscon G., Paci P. (2019). MIENTURNET: An Interactive Web Tool for microRNA-Target Enrichment and Network-Based Analysis. BMC Bioinform..

[31] Shannon P., Markiel A., Ozier O., Baliga N.S., Wang J.T., Ramage D., Amin N., Schwikowski B., Ideker T. (2003). Cytoscape: A Software Environment for Integrated Models of Biomolecular Interaction Networks. Genome Res..

[32] Li J., Han X., Wan Y., Zhang S., Zhao Y., Fan R., Cui Q., Zhou Y. (2018). TAM 2.0: Tool for MicroRNA Set Analysis. Nucleic Acids Res..

[33] Aparicio-Puerta E., Hirsch P., Schmartz G.P., Kern F., Fehlmann T., Keller A. (2023). miEAA 2023: Updates, New Functional microRNA Sets and Improved Enrichment Visualizations. Nucleic Acids Res..

