# Supplementary material for: An Exploratory Biomarker Study of First-Trimester Circulating miRNAs Associated with Later Gestational Diabetes Mellitus
Source: Int J Mol Sci. 2026 Feb 17;27(4):1920. doi: 10.3390/ijms27041920 (PMC12940769; doi:10.3390/ijms27041920)
Supplement: Supplementary file 1 [file ijms-27-01920-s001.zip › Table_S1_Clinical_Characteristics_of_Serum_Pools.pdf]

Table S1. Summary of the clinical characteristics of the three pooled first-trimester plasma samples (CON, n-APO, APO).

Variable	CON (n=10)	n-APO (n=10)	APO (n=10)
Age (years), mean ± SD	28.3 ± 1.34	25.6 ± 3.20	29.9 ± 7.02
BMI (kg/m²), mean ± SD	29.9 ± 3.83	29.9 ± 4.13	34.2 ± 4.38
First-degree family history, %	60%	60%	60%
OGTT fasting (mg/dL), median (IQR)	80.0 (7.75)	83.0 (14.25)	95.5 (19.75)
OGTT 1-hour (mg/dL), median (IQR)	131.5 (21.0)	173.0 (37.25)	186.5 (24.75)
OGTT 2-hour (mg/dL), median (IQR)	119.0 (29.25)	124.0 (29.75)	168.0 (11.0)

Summary of the clinical characteristics of the three pooled first-trimester plasma samples analyzed by small RNA sequencing: normoglycemic controls (CON), GDM cases without adverse outcomes (n-APO), and GDM cases with adverse pregnancy outcomes (APO). Values are shown as mean ± SD for age and BMI, and as median (IQR) for OGTT glucose levels. First-degree family history represents the percentage of women reporting at least one first-degree relative with diabetes.
