# Supplementary material for: An Exploratory Biomarker Study of First-Trimester Circulating miRNAs Associated with Later Gestational Diabetes Mellitus
Source: Int J Mol Sci. 2026 Feb 17;27(4):1920. doi: 10.3390/ijms27041920 (PMC12940769; doi:10.3390/ijms27041920)
Supplement: Supplementary file 1 [file ijms-27-01920-s001.zip › Table_S2_Functional_Categories_miRNA_Pathways_MAIN.pdf]

Table S2. Enriched functional categories and representative miRNAs associated with insulin-related and metabolic pathways, based on set-level annotation analyses

Category (Database)	Subcategory (Pathway / Process)	Direction (↑ / ↓ miRNAs)	Adjusted p-value (FDR)	Representative miRNAs	Validated Targets (miRTarBase)	Predicted Targets (TargetScan)	Functional annotation
GO Biological Process (miRTarBase)	Regulation of glucagon secretion (GO:0070092)	↓	0.004	hsa-miR-25-3p ↑, hsa-let-7c-5p ↓, hsa-miR-92a-3p ↑, hsa-let-7f-5p ↓, hsa-miR-29a-3p ↑, hsa-miR-29c-3p ↑	*ATP2A2*, *ITPR1*, *RYR2*	*ATP2A2*, *ITPR1*, *KCNK3*	Ca^{2+}-dependent signaling via ITPR1 and RYR2 controls glucagon exocytosis from α-cells. Altered miRNA regulation may impair glucagon suppression in hyperglycemia, aggravating hepatic glucose production.
GO Biological Process (miRTarBase)	Endoplasmic reticulum ion homeostasis (GO:0032469)	↓	0.006	hsa-miR-25-3p ↑, hsa-let-7f-5p ↓, hsa-let-7c-5p ↓, hsa-miR-92a-3p ↑, hsa-miR-29a-3p ↑, hsa-miR-29c-3p ↑	*ATP2A2*, *CALM1*, *CALM3*, *ITPR1*, *RYR2*	*ATP2A2*, *CALM1*, *CALM3*, *ITPR1*	Disturbance in ATP2A2- and ITPR1-mediated Ca^{2+} handling disrupts ER–cytoplasm calcium exchange, causing ER stress and β-cell apoptosis—key features of type 2 diabetes and gestational insulin resistance.

KEGG Pathway	PI3K–AKT–mTOR signaling	↑	0.056	hsa-miR-29a-3p ↑, hsa-miR-29c-3p ↑, hsa-miR-92a-3p ↑, hsa-miR-182-5p ↑, hsa-miR-146a-5p ↑, hsa-miR-146b-5p ↑	*AKT1*, *AKT2*, *AKT3*, *FOXO1*, *MTOR*, *PIK3R1*, *PTEN*, *TSC1*	*PIK3CA*, *PIK3R1*, *PTEN*, *TSC1*	Activation of the PI3K/AKT/mTOR pathway promotes glucose uptake and protein synthesis while inhibiting apoptosis. Dysregulation leads to insulin resistance through altered phosphorylation of AKT and downstream effectors (e.g., FOXO1, TSC1/TSC2, mTORC1).
Reactome	Insulin receptor signaling cascade	↑	0.049	hsa-miR-29a-3p ↑, hsa-miR-29c-3p ↑, hsa-miR-25-3p ↑, hsa-miR-92a-3p ↑, hsa-miR-183-5p ↑, hsa-miR-192-5p ↑	*MAPK1*, *PIK3R1*	*IRS2*, *MAPK1*, *PIK3CA*, *PIK3R1*	Binding of insulin to INSR activates IRS1/2 and PI3K/AKT, leading to GLUT4 translocation and glycogen synthesis. Impaired signaling diminishes glucose transport and contributes to systemic insulin resistance.
GO Biological Process	Regulation of	↓	0.018	hsa-let-7a-5p ↓, hsa-let-7c-5p ↓, hsa-let-	*SYT1*	*CACNA1C*, *SYT1*	Calcium influx through CACNA1C and vesicle fusion

insulin secretion	7f-5p ↓, hsa-miR-25-3p ↑, hsa-miR-29a-3p ↑, hsa-miR-92a-3p ↑	mediated by SYT1 regulate insulin exocytosis. Altered miRNA expression reduces β-cell excitability and secretory capacity, leading to defective insulin release.
