# Supplementary material for: An Exploratory Biomarker Study of First-Trimester Circulating miRNAs Associated with Later Gestational Diabetes Mellitus
Source: Int J Mol Sci. 2026 Feb 17;27(4):1920. doi: 10.3390/ijms27041920 (PMC12940769; doi:10.3390/ijms27041920)
Supplement: Supplementary file 1 [file ijms-27-01920-s001.zip › Table_S3_Target-genes-regulated-by-multiple-relevant-miRNAs.pdf]

Table S3. Target genes annotated as shared targets of multiple circulating miRNAs. This table lists all target genes curated in miRTarBase that are annotated as targets of two or more of the 18 circulating miRNAs identified in this study. Columns include the gene name, the number of annotated regulating miRNAs, the identity of these miRNAs, and the rank of each miRNA based on Table 1.

Gen	Number of regulating miRNAs	Regulating miRNAs	Regulating miRNA Ranks in Table 1
PTEN	6	miR-106b-3p, miR-182-5p, miR-25-3p, miR-29a-3p, miR-29c-3p, miR-92a-3p	1, 2, 4, 5, 10, 11
IL6	6	let-7a-5p, let-7c-5p, let-7f-5p, miR-146a-5p, miR-146b-5p, miR-451a	3, 8, 9, 15, 16, 17
CCND2	5	let-7a-5p, miR-146a-5p, miR-182-5p, miR-29a-3p, miR-29c-3p	1, 4, 8, 10, 17
BCL2	5	miR-182-5p, miR-192-5p, miR-29a-3p, miR-29c-3p, miR-451a	1, 3, 4, 7, 10
KLF4	4	miR-25-3p, miR-29a-3p, miR-29c-3p, miR-92a-3p	1, 2, 4, 5
CDKN1A	4	let-7a-5p, miR-146a-5p, miR-146b-5p, miR-182-5p	8, 9, 10, 17
ITGB1	4	miR-183-5p, miR-192-5p, miR-29a-3p, miR-29c-3p	1, 4, 6, 7
DICER1	4	let-7a-5p, let-7c-5p, miR-192-5p, miR-29a-3p	1, 7, 15, 17
MYC	4	let-7a-5p, let-7c-5p, miR-29a-3p, miR-451a	1, 3, 15, 17
GSK3B	3	miR-182-5p, miR-183-5p, miR-29a-3p	1, 6, 10
CCND1	3	let-7f-5p, miR-146a-5p, miR-29a-3p	1, 8, 16
EGFR	3	let-7a-5p, miR-146a-5p, miR-146b-5p	8, 9, 17
SMAD4	3	miR-146a-5p, miR-182-5p, miR-183-5p	6, 8, 10
RECK	3	miR-182-5p, miR-183-5p, miR-25-3p	5, 6, 10

FBXW7	3	miR-182-5p, miR-25-3p, miR-92a-3p	2, 5, 10
MMP2	3	miR-29a-3p, miR-29c-3p, miR-451a	1, 3, 4
CDK6	3	let-7a-5p, miR-29a-3p, miR-29c-3p	1, 4, 17
AKT1	3	miR-192-5p, miR-196b-5p, miR-451a	3, 7, 13
STAT3	3	let-7a-5p, let-7c-5p, miR-92a-3p	2, 15, 17
MPL	3	let-7a-5p, let-7c-5p, let-7f-5p	15, 16, 17
ITGB3	3	let-7a-5p, let-7c-5p, miR-192-5p	7, 15, 17
LAMC2	3	miR-146a-5p, miR-29a-3p, miR-29c-3p	1, 4, 8
IRAK1	2	miR-146a-5p, miR-146b-5p	8, 9
EZH2	2	let-7a-5p, miR-25-3p	5, 17
FGA	2	miR-29a-3p, miR-29c-3p	1, 4
MCL1	2	miR-29a-3p, miR-29c-3p	1, 4
FBN1	2	miR-29a-3p, miR-29c-3p	1, 4
FAS	2	miR-146a-5p, miR-196b-5p	8, 13
NRAS	2	let-7a-5p, let-7c-5p	15, 17
RHOA	2	miR-146a-5p, miR-483-5p	8, 14
FGG	2	miR-29a-3p, miR-29c-3p	1, 4
ERBB4	2	miR-146a-5p, miR-146b-5p	8, 9
NFKB1	2	miR-146a-5p, miR-146b-5p	8, 9
MTOR	2	let-7c-5p, miR-196b-5p	13, 15
DNMT3A	2	miR-29a-3p, miR-29c-3p	1, 4
DNMT1	2	miR-29a-3p, miR-92a-3p	1, 2
FGB	2	miR-29a-3p, miR-29c-3p	1, 4
RARB	2	miR-146a-5p, miR-146b-5p	8, 9
MIF	2	miR-146a-5p, miR-451a	3, 8
MYCN	2	miR-29a-3p, miR-29c-3p	1, 4

KAT2B	2	miR-25-3p, miR-92a-3p	2, 5
FOXO1	2	miR-182-5p, miR-183-5p	6, 10
FOXO3	2	miR-182-5p, miR-29a-3p	1, 10
LRP6	2	miR-183-5p, miR-29c-3p	4, 6
ITGA6	2	miR-29a-3p, miR-29c-3p	1, 4
HMGA2	2	let-7a-5p, let-7c-5p	15, 17
HMGCR	2	miR-29a-3p, miR-29c-3p	1, 4
DHFR	2	miR-192-5p, miR-25-3p	5, 7
NUMB	2	let-7c-5p, miR-146a-5p	8, 15
PER1	2	miR-29a-3p, miR-29c-3p	1, 4
PDGFRB	2	miR-29a-3p, miR-29c-3p	1, 4
PDCD4	2	miR-182-5p, miR-183-5p	6, 10
IGF2BP1	2	let-7a-5p, miR-196b-5p	13, 17
MAP2K4	2	miR-25-3p, miR-92a-3p	2, 5
DNMT3B	2	miR-29a-3p, miR-29c-3p	1, 4
CDKN1B	2	miR-182-5p, miR-192-5p	7, 10
SNAI2	2	miR-182-5p, miR-183-5p	6, 10
TLR4	2	miR-146a-5p, miR-146b-5p	8, 9
CASP3	2	let-7a-5p, let-7c-5p	15, 17
TIAM1	2	miR-182-5p, miR-29c-3p	4, 10
TRAF6	2	miR-146a-5p, miR-146b-5p	8, 9
TGFBR2	2	miR-196b-5p, miR-92a-3p	2, 13
BMI1	2	miR-183-5p, miR-192-5p	6, 7
BCL2L11	2	miR-25-3p, miR-92a-3p	2, 5
TDG	2	miR-29a-3p, miR-29c-3p	1, 4
TRIM71	2	let-7a-5p, let-7c-5p	15, 17

BACE1	2	miR-29a-3p, miR-29c-3p	1, 4
CDC42	2	miR-29a-3p, miR-29c-3p	1, 4
CDC7	2	miR-192-5p, miR-29a-3p	1, 7
CDH1	2	miR-25-3p, miR-92a-3p	2, 5
TNFAIP3	2	let-7a-5p, miR-29a-3p	1, 17
TNFRSF10B	2	let-7a-5p, let-7c-5p	15, 17
COL10A1	2	miR-29a-3p, miR-29c-3p	1, 4
VEGFA	2	miR-29a-3p, miR-29c-3p	1, 4
SIRT1	2	miR-29c-3p, miR-92a-3p	2, 4
COL1A2	2	miR-29a-3p, miR-29c-3p	1, 4
CREB5	2	miR-182-5p, miR-29c-3p	4, 10
AKT2	2	miR-29a-3p, miR-29c-3p	1, 4
COL3A1	2	miR-29a-3p, miR-29c-3p	1, 4
AKT3	2	miR-29a-3p, miR-29c-3p	1, 4
COL4A1	2	miR-29a-3p, miR-29c-3p	1, 4
ALCAM	2	miR-192-5p, miR-483-5p	7, 14
COL4A2	2	miR-29a-3p, miR-29c-3p	1, 4
COL5A2	2	miR-29a-3p, miR-29c-3p	1, 4
XIAP	1	miR-192-5p	7
TRAF4	1	miR-29a-3p	1
MYH9	1	let-7f-5p	16
UVRAG	1	miR-183-5p	6
NOTCH2	1	miR-146a-5p	8
TSC1	1	miR-451a	3
ULBP2	1	miR-182-5p	10
MYLIP	1	miR-92a-3p	2
NF2	1	let-7a-5p	17
UQCRFS1	1	miR-182-5p	10
SEMA4C	1	miR-25-3p	5
TGFBR1	1	let-7c-5p	15
PCGF5	1	miR-92a-3p	2
SRF	1	miR-483-5p	14

RRM2	1	let-7a-5p	17
ROBO1	1	miR-29a-3p	1
SLC5A5	1	miR-146b-5p	9
RNASEL	1	miR-29a-3p	1
SMAD7	1	miR-25-3p	5
REV3L	1	miR-25-3p	5
RDX	1	miR-196b-5p	13
SP1	1	miR-29c-3p	4
SRSF10	1	miR-29c-3p	4
PDGFRA	1	miR-146b-5p	9
RAD21	1	miR-92a-3p	2
PTGES2	1	miR-146a-5p	8
PPM1D	1	miR-29a-3p	1
PKM	1	let-7a-5p	17
PIK3R1	1	miR-29a-3p	1
SETDB1	1	miR-29a-3p	1
PFN1	1	miR-182-5p	10
THBS1	1	miR-182-5p	10
ACVR2B	1	miR-192-5p	7
HOXA9	1	miR-196b-5p	13
MEIS1	1	miR-196b-5p	13
CASP8	1	let-7a-5p	17
CUL5	1	miR-192-5p	7
CPEB4	1	miR-29a-3p	1
CPEB3	1	miR-29a-3p	1
COX2	1	miR-146a-5p	8
CNOT6	1	miR-29c-3p	4
CKB	1	miR-483-5p	14
CDKN2D	1	miR-451a	3
CDK4	1	miR-29a-3p	1
CDC25A	1	let-7c-5p	15
CD276	1	miR-29a-3p	1
CCNT2	1	miR-29a-3p	1
CCNA2	1	miR-146a-5p	8
CASP7	1	miR-146a-5p	8
MDM2	1	miR-25-3p	5
CADM1	1	miR-182-5p	10
BTRC	1	miR-183-5p	6
BRCA2	1	miR-146a-5p	8
BRCA1	1	miR-146a-5p	8
BMPR2	1	miR-92a-3p	2
BARD1	1	miR-182-5p	10
AURKB	1	let-7a-5p	17
ATP2A2	1	miR-25-3p	5
ATG9A	1	miR-29a-3p	1
ATF1	1	miR-182-5p	10
ALDH5A1	1	miR-29a-3p	1
AHR	1	miR-29a-3p	1
CXCL8	1	miR-146a-5p	8

CYP2C19	1	miR-29a-3p	1
DSC2	1	miR-25-3p	5
DUSP1	1	miR-146a-5p	8
MAPK1	1	miR-451a	3
MAP3K1	1	miR-451a	3
LMNB2	1	miR-192-5p	7
LATS2	1	miR-25-3p	5
LAMC1	1	miR-29c-3p	4
KRAS	1	let-7a-5p	17
ITGA5	1	miR-92a-3p	2
IL6R	1	miR-451a	3
IGF1R	1	let-7c-5p	15
IFNAR1	1	miR-29a-3p	1
ICAM1	1	miR-146a-5p	8
ADAM10	1	miR-451a	3
HOXA10	1	miR-192-5p	7
HIPK1	1	miR-92a-3p	2
HDAC2	1	miR-92a-3p	2
GLUL	1	miR-29a-3p	1
GAPDH	1	miR-29c-3p	4
FZD4	1	miR-29c-3p	4
FRAT2	1	miR-29c-3p	4
FOS	1	miR-196b-5p	13
FASN	1	miR-532-5p	12
FANCM	1	miR-146a-5p	8
EZR	1	miR-183-5p	6
EWSR1	1	let-7a-5p	17
EGR1	1	miR-183-5p	6
ZNRF3	1	miR-146b-5p	9

Notes:

● All miRNA–gene interactions correspond exclusively to experimentally validated records curated in miRTarBase.

● "Number of miRNAs" refers to how many of the 18 circulating miRNAs are annotated as targeting each gene.

● "Regulating miRNA rank in Table 1" indicates the position assigned to each miRNA in Table 1.
